# Can we treat bladder cancer with intravesical Bacillus Calmette-Guerin in patients with prior tuberculosis infection? A population-based cohort study

**DOI:** 10.1186/s12894-020-00642-1

**Published:** 2020-07-08

**Authors:** Che-Wei Hsu, Yi-Chun Chiu, Hsiao-Yun Hu, Yu-Hua Fan, Shih-Chi Hong, Wei-Ming Cheng

**Affiliations:** 1 Division of Urology, Department of Surgery, Taipei City Hospital Zhongxiao Branch, Taipei, Taiwan; 2 Division of Urology, Department of Surgery, Taipei City Hospital Heping Fuyou Branch, Taipei, Taiwan; 3 Department of Urology, School of Medicine, National Yang-Ming University, Taipei, Taiwan; 4 Department of Education and Research, Taipei City Hospital, Taipei, Taiwan; 5 Institute of Public Health, National Yang-Ming University, Taipei, Taiwan; 6 Department of Urology, Taipei Veterans General Hospital, Taipei, Taiwan

**Keywords:** Bladder cancer, Urothelial carcinoma, Intravesical immunotherapy, Bacillus Calmette-Guerin instillation, Tuberculosis infection

## Abstract

**Background:**

Intravesical bacillus Calmette-Guerin (BCG) therapy is the treatment of choice for patients with T1 or high-grade superficial bladder cancer or those with carcinoma in situ after transurethral resection. A personal history of tuberculosis infection has been viewed as a relative contraindication for BCG therapy, because it may increase the risk of complications or decrease the treatment effectiveness. We determined the safety and efficacy of intravesical BCG treatment for patients with prior tuberculosis infection by analyzing the data obtained from the National Health Insurance Research Database in Taiwan.

**Methods:**

We included patients who were newly diagnosed with bladder cancer from 2000 to 2009 and who received adjuvant intravesical BCG therapy within 3 months after the surgery. We excluded those who developed upper urinary tract cancer during the study period. Disease recurrence, disease progression, and major adverse effects were compared between patients with and without a prior diagnosis of tuberculosis infection until December 31, 2011.

**Results:**

Among the 3915 patients included, 187 (4.8%) had been previously diagnosed with tuberculosis infection. The proportion of men (84.0% versus 76.9%) and older patients was higher in the group with a prior tuberculosis infection than in those without a prior tuberculosis infection. Significant differences in disease recurrence (20.3% versus 22.8%; hazard ratio [HR], 0.87; 95% confidence interval [CI], 0.63–1.21, *p* = 0.404) or disease progression (10.2% versus 12.8%, HR, 0.74; 95% CI, 0.46–1.17, *p* = 0.191) were not observed between the two groups. None of the patients with a prior tuberculosis infection had severe urinary tract infections, whereas four (0.1%) patients without such an infection developed severe urinary tract infections.

**Conclusion:**

A prior tuberculosis infection did not affect the treatment efficacy or safety of intravesical BCG treatment. The efficacy and safety of intravesical BCG therapy are comparable between bladder cancer patients with and without prior tuberculosis infections.

## Background

Bladder cancer is the 10th most common cancer worldwide, with an estimated 549,000 new cases and 200,000 deaths each year [1]. Nearly 70% of cases are superficial or non-muscle-invasive bladder cancers (NMIBCs) at the initial presentation [2]. The initial treatment for NMIBC includes complete transurethral resection (TUR), followed by adjuvant intravesical immunotherapy or chemotherapy to minimize the possibility of recurrence [3, 4]. Bacillus Calmette-Guérin (BCG), the agent used as an intravesical immunotherapy, is an attenuated mycobacterial vaccine for tuberculosis (TB) that has been proven to have an antitumor activity in several different cancers, including urothelial carcinoma [5]. The BCG therapy results in a massive local immune response characterized by an influx of granulocytes and mononuclear and dendritic cells induced by IL-6 and other cytokines in the urine and bladder wall [6]. It has been shown that the BCG therapy is more effective than other intravesical chemotherapy regimens in preventing disease recurrence after the TUR surgery, especially in those with high-grade urothelial carcinoma or carcinoma in situ [7]. However, it also possesses various adverse effects, including local irritation or cystitis, systemic fever, general malaise, or even sepsis [8]. As a result, BCG therapy is contraindicated in patients with traumatic catheterization, profound urinary tract inflammation, and gross hematuria. A personal history of TB infection has also been viewed as a relative contraindication, because it might increase the risk of complications, although this has not been supported by any clinical data. In the present study, we aimed to provide evidence for this hypothesis by evaluating the efficacy and adverse effects of intravesical BCG treatment of NMIBC in patients with a prior TB infection, who were registered in the National Health Insurance Research Database (NHIRD) in Taiwan.

## Methods

### Data source

The National Health Insurance (NHI) program was established on March 1, 1995, and more than 99.9% of Taiwan’s residents are enrolled in this program. The NHIRD, containing registration files and original claims data for reimbursement, is provided by the National Health Research Institutes (NHRI) to scientists in Taiwan for research. One of its databases, the Longitudinal Health Insurance Database 2000 (LHID2000), contains all the original claims data of 100,000 beneficiaries randomly sampled from the 2000 Registry of Beneficiaries of the NHIRD [9]. The data includes patients’ ambulatory and hospitalization orders, procedures, and medications. All data are de-identified before being sent to the NHRI for database construction to prevent identification of patients or care providers and are further de-identified before being released to each researcher. All researchers are required to sign a written agreement declaring that they have no intention to obtain information that could potentially violate the privacy of patients or care providers. Applicants must follow the related regulations of NHRI and are reviewed for approval of data release.

### Study subjects

**Fig. 1:**
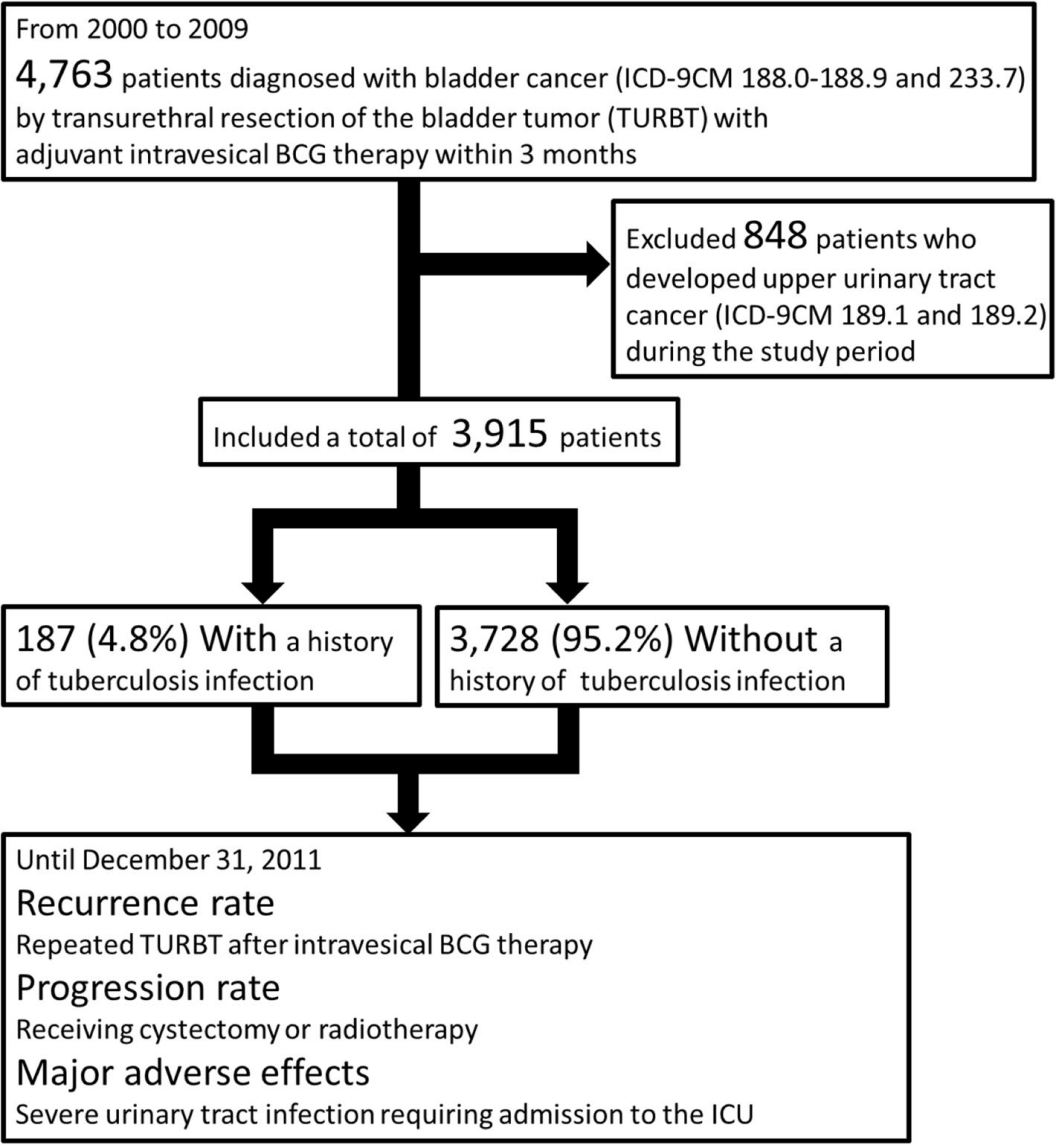
Study Protocol of Patient Inclusion and Exclusion and the Number of Patients

Patients without a history of bladder cancer in the past 2 years but who were diagnosed with bladder cancer (ICD-9CM 188.0–188.9 and 233.7) by TUR from 2000 to 2009 and who underwent adjuvant intravesical BCG therapy within 3 months after surgery were identified from the LHID2000. Patients with a history of upper urinary tract cancer or who developed upper urinary tract cancer (ICD-9CM 189.1 and 189.2) during the follow-up period were excluded from the analysis (Fig. 1).

### Outcome measures

The primary endpoints, including disease recurrence, disease progression, and episodes of major adverse effects in each patient within 2 years after the first TUR surgery, were compared between patients with a prior diagnosis of TB infection (ICD-9CM 010–018) and those without such diagnosis. Disease recurrence was defined as repeated TUR within 3 months since the initial TUR to avoid the contamination of second-look TUR surgery. Patients receiving cystectomy or radiotherapy for bladder cancer after undergoing intravesical BCG therapy were considered as having a disease progression. Patients with severe urinary tract infections (ICD-9CM 599.0, 038, 590, and 595) requiring admission to the intensive care unit were considered as having an episode of major adverse effects. We also compared the number of instillations within 3 months since the initiation of the first intravesical therapy between patients with and without a prior TB infection.

### Statistical analysis

The chi-square and Fisher’s exact tests were used to measure the significant differences in the categorical variables between the two groups, whereas the Wilcoxon rank sum test was used to compare the endpoints. Disease recurrence and disease progression were estimated using the Kaplan-Meier method and compared using the log-rank test. Univariate and multivariable analyses were performed using the Cox proportional hazards models. A Poisson regression model was used to assess the number of instillations. We performed all analyses using the SAS System for Windows (version 9.4, SAS Institute Inc., Cary, NC, USA).

## Results

**Fig. 2:**
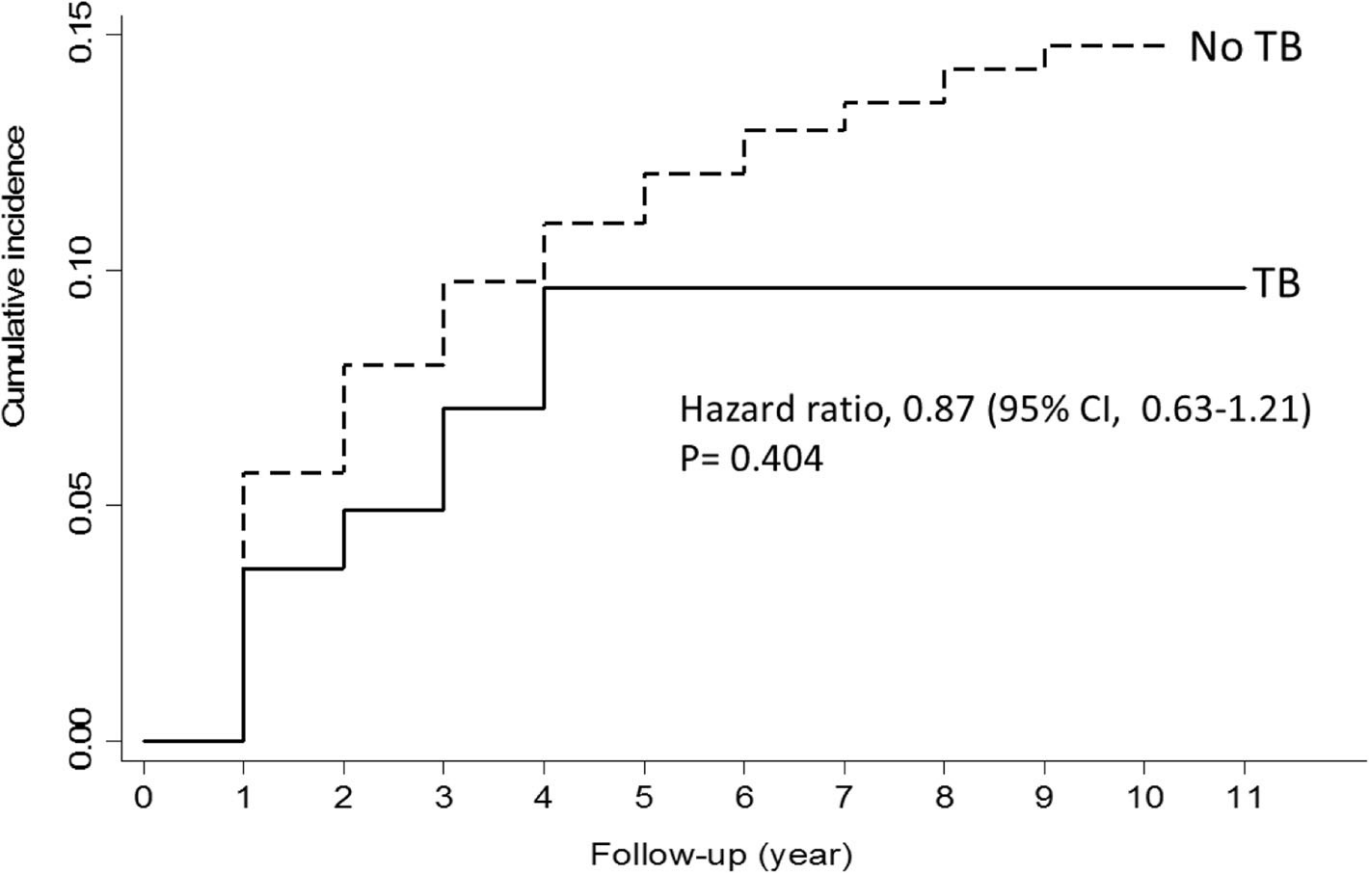
Kaplan-Meier Estimates of Disease Recurrence

**Fig. 3:**
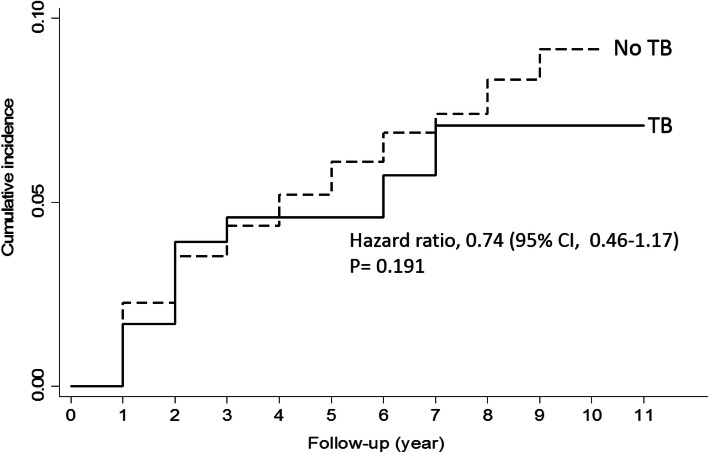
Kaplan-Meier Estimates of Disease Progression

A total of 4763 patients underwent TUR and adjuvant intravesical BCG therapy for bladder cancer for the first time from 2000 to 2009. A total of 848 patients had synchronous or metachronous upper urinary tract cancers and were excluded from the analysis. Among the 3915 patients included in the analysis, 187 (4.8%) had a prior TB infection (Fig. 1). Compared to patients without a prior TB infection, the patients with a prior TB infection were more likely to be male (84.0% versus 76.9%, *p* = 0.025) and older (*p* < 0.001; Table 1). There were no significant differences in disease recurrence (20.3% versus 22.8%, hazard ratio [HR] 0.87; 95% confidence interval [CI], 0.63–1.21, *p* = 0.404; Fig. 2) or disease progression (10.2% versus 12.8%, HR, 0.74; 95% CI, 0.46–1.17, *p* = 0.191; Fig. 3). None of the patients with prior TB infection had severe urinary tract infections, whereas four patients without prior TB infection (0.1%) developed this major adverse effect after intravesical BCG therapy (*p* > 0.999). In terms of the number of instillations, patients with a prior TB infection had significantly fewer instillation episodes (*p* = 0.017) than those without such an infection; however, the difference became non-significant after adjusting for sex and age using the Poisson regression test (β = − 0.113, *p* = 0.290; Table 2).

**Table 1:** Demographics of patients with and without prior TB infection who received adjuvant intravesical BCG therapy

Prior TB infection	Yes		No		***P***-value
Numbers	187	(4.8%)	3728	(95.2%)	
Sex					0.025
Female	30	(16.0%)	860	(23.1%)	
Male	157	(84.0%)	2868	(76.9%)	
Age					< 0.001
< 50 years	7	(3.7%)	371	(10.0%)	
50–64 years	32	(17.1%)	1141	(30.6%)	
65–74 years	62	(33.2%)	1133	(30.4%)	
≧75 years	86	(46.0%)	1083	(29.0%)	
Disease recurrence	38	(20.3%)	851	(22.8%)	
Times of instillation before disease recurrence	6	(1–9)	6	(1–48)	0.551
Disease progression (cystectomy + radiotherapy)	19	(10.2%)	477	(12.8%)	
Partial or radical cystectomy	2	(1.1%)	94	(2.5%)	
Times of instillation before cystectomy	9	(6–12)	6	(1–21)	0.281
Radiotherapy	18	(9.6%)	413	(11.1%)	
Times of instillation before radiotherapy	6	(1–18)	6	(1–58)	0.550
Severe urinary tract infection	0	(0.0%)	4	(0.1%)	0.999
Total times of instillation within 3 months	6	(1–8)	6	(1–9)	0.017^*^

BCG, bacillus Calmette-Guerin; TB, tuberculosis

**Table 2:** Multivariable analyses of the total times of instillation within 3 months

Variable	ß	SE	P-value
Sex			
Male	0.191	0.056	< 0.001
Age			
50–64 years	0.254	0.098	0.010
65–74 years	0.493	0.096	< 0.001
≧75 years	0.533	0.096	< 0.001
With prior TB vs. without prior TB	−0.113	0.107	0.290

TB, tuberculosis

## Discussion

By analyzing a nationwide population-based database, we found that a prior TB infection had no impact on the efficacy or safety of intravesical BCG therapy, in terms of disease recurrence, disease progression, and incidence of severe urinary tract infection. Although the patients with a prior TB infection were more likely to have a delay in their instillation schedule than those without such an infection, no difference was found between the two groups after adjusting for age and sex. In brief, prior TB infections should not be a contraindication for adjuvant intravesical BCG therapy in patients with NMIBC.

Currently, TB is the leading cause of death from a single infectious pathogen worldwide, and approximately 10.0 million people have developed TB globally [10]. More than 50% of the global TB cases are found in Southeastern Asia and the Western Pacific [11]. Therefore, many patients with bladder cancer would benefit from BCG treatment, if a prior TB infection is no longer a contraindication for this therapy. Bohle at el. found that the cystectomy rate, as well as the time to cystectomy, of patients with carcinoma in situ in the urinary bladder was significantly decreased when treated with BCG (11% vs. 55% for controls), [12]. As in the present study, patients with a prior TB infection were more likely to be older and might not be suitable candidates for radical cystectomy if they were diagnosed with high-risk NMIBC or carcinoma in situ. Interventions to minimize the possibility of disease recurrence or disease progression after TUR are important for these patients. Our study showed that adjuvant intravesical BCG therapy was effective and safe in this population.

Increased local and systemic inflammatory responses are observed after intravesical instillation of BCG. The upregulation of cytokines, including interleukin-1 (IL-1), IL-2, IL-5, IL-6, IL-8, IL-10, IL-12, and IL-18, indicates a T-helper type-1 (Th1) response, activating cell-mediated cytotoxicity to the urothelial cancer cells [13]. Prior exposure to the antigens of *Mycobacterium tuberculosis* should accelerate and reinforce the immune response. A study on mice showed that immunization with BCG vaccine subcutaneously before the establishment of orthotopic bladder cancer evoked a robust inflammatory response, accelerated the entry of T cells into the bladder, and reinforced its antitumor effect after administration of intravesical BCG. Niwa et al. recruited 55 NMIBC patients with positive and negative purified protein derivative (PPD) tests, respectively. Those with positive test results had better recurrence-free survival after treatment with adjuvant intravesical BCG [14]. Another study included 288 NMIBC patients subjected to the PPD skin test. The reactions before BCG therapy could predict the therapeutic effects of BCG therapy and potentially the major BCG-related side effects [15]. However, the determination and interpretation of the PPD test are subjective, depending on the size of the induration after injection. Patients with a history of TB infection may have a false-negative result in the PPD test, and a false-positive result may occur in certain conditions, such as BCG vaccination or infection with non-tuberculosis *Mycobacterium*. The impact of BCG vaccination on later adjuvant intravesical BCG therapy in humans remains unknown. One study suggested that a reduced dosage of BCG instillation acts better in European patients than in North American patients. The authors postulated that BCG vaccination in some European countries may account for such phenomenon [16]. In Taiwan, BCG vaccination has been routinely performed since 1977 [17], and most of the patients in our study had not previously received BCG vaccination. It remains safe and effective to treat patients with a prior TB infection with intravesical BCG, according to our findings.

The recurrence and progression rates were estimated to be approximately 20 and 10%, respectively, similar to the results of other studies. Lamm et al. reported that the recurrence rates of 20–33% after treatment with adjuvant intravesical BCG and TUR compared to that after treatment with TUR alone [18]. Other studies showed a substantial efficacy of BCG treatment, and the progression rate was approximately 11–24% [19, 20]. This suggests that the application of the codes of TUR surgery after at least three rounds of intravesical BCG instillation was an acceptable method for identifying patients with disease recurrence, whereas the codes for cystectomy and radiation therapy could represent disease progression. In the literature, the incidence of sepsis after intravesical BCG therapy was approximately 0.4% [21], consistent with our study findings.

There are several limitations in our study. First, this was a retrospective study based on a nationwide insurance database. The database did not include pathological reports; thus, we were unable to stratify the differences in the responses of patients with NMIBC according to the different pathological findings in this study. Second, Taiwan’s National Health Insurance was established in 1995, and patients diagnosed and treated with TB infections before the year 1995 may not always be included in the NHIRD. As a result, we applied the loose criterion, that is, any suspicious diagnostic codes for TB infection, rather than the definite antibiotics used for TB treatment, were used to identify those with prior TB infections. Third, some reports suggested that the different BCG strains would possess varying anti-tumor effects [22]. However, this is controversial, and it is impossible to identify the strains of the BCG used by each pharmaceutical company in the NHIRD. Fourth, some patient variables, such as smoking status, socioeconomic status, and comorbidities were, not discussed in our study. Nevertheless, this study remains worthy, because, to the best of our knowledge, this is the largest study that investigated the safety and efficacy of intravesical BCG therapy in patients with a prior TB infection. A study including more patients that prospectively records the recurrence, progression, as well as major and minor adverse effects can further identify the impact of prior TB infection on the treatment efficacy and safety of intravesical BCG therapy and help clarify the mechanism behind this intravesical immunotherapy.

## Conclusions

A history of prior TB infection should not be a contraindication for adjuvant intravesical BCG immunotherapy, because it does not affect the treatment efficacy or safety in patients with non-muscle invasive bladder cancer. A prospective study including more patients is warranted to verify this finding.

## Data Availability

The datasets supporting the conclusions of this article are available in the National Health Insurance Research Database, Taiwan [https://nhird.nhri.org.tw/en/index.html]. Only citizens of the Republic of China who fulfill the requirements of conducting research projects are eligible to apply for the National Health Insurance Research Database (NHIRD). The use of NHIRD is limited to research purposes only. Applicants must follow the Computer-Processed Personal Data Protection Law (http://www.winklerpartners.com/?p=987) and related regulations of National Health Insurance Administration and NHRI (National Health Research Institutes), and an agreement must be signed by the applicant and his/her supervisor upon application submission. All applications are reviewed for approval of data release.

## Abbreviations

NHI: National Health Insurance
NHRI: National Health Research Institutes
NHIRD: National Health Insurance Research Database
NMIBC: Non-muscle-invasive bladder cancer
PPD: Purified protein derivative
TB: Tuberculosis
TUR: Transurethral resection

## References

[1] Bray F, Ferlay J, Soerjomataram I, Siegel RL, Torre LA, Jemal A (2018). Global cancer statistics 2018: GLOBOCAN estimates of incidence and mortality worldwide for 36 cancers in 185 countries. CA Cancer J Clin.

[2] Kamat AM, Hahn NM, Efsttathiou JA, Lerner SP, Malmström PU, Choi W (2016). Bladder cancer. Lancet..

[3] Babjuk M, Bohle A, Burger M, Capoun O, Cohen D, Compérat EM (2017). EAU guidelines on non–muscle-invasive urothelial carcinoma of the bladder: update 2016. Eur Urol.

[4] Clark PE, Spiess PE, Agarwal N, Bangs R, Boorjian SA, Buyyounouski MK (2016). NCCN guidelines insights: bladder cancer, version 2.2016. J Natl Compr Cancer Netw.

[5] Morales A, Eidinger D, Bruce AW (1976). Intracavitary bacillus Calmette-Guerin in the treatment of superficial bladder tumors. J Urol.

[6] Shen Z, Shen T, Wientjes MG, O’Donnell MA, Au JLS (2008). Intravesical treatments of bladder cancer: review. Pharm Res.

[7] Hall MC, Chang SS, Dalbagni G, Pruthi RS, Seigne JD, Skinner EC (2007). Guideline for the management of nonmuscle invasive bladder cancer (stages ta, T1, and tis): 2007 update. J Urol.

[8] van der Meijden AP, Sylvester RJ, Oosterlinck W, Hoeltl W, Bono AV, EORTC Genito-Urinary Tract Cancer Group. Maintenance bacillus Calmette-Guerin for Ta T1 bladder tumors is not associated with increased toxicity: results from a European Organisation for Research and Treatment of Cancer Genito-Urinary Group Phase III Trial. Eur Urol. 2003;44:429–34. 10.1016/s0302-2838(03)00357-9 14499676

[9] Chen Y-C, Yeh H-Y, Wu J-C, Haschler I, Chen T-J, Wetter T (2011). Taiwan’s National Health Insurance Research Database: administrative health care database as study object in bibliometrics. Scientometrics..

[10] World Health Organization. Global tuberculosis report 2018. https://reliefweb.int/sites/reliefweb.int/files/resources/9789241565646-eng.pdf. Accessed 23 Dec 2019.

[11] World Health Organization. WHO report 2009: global tuberculosis control–epidemiology, strategy, financing https://reliefweb.int/report/world/global-tuberculosis-control-2009-epidemiology-strategy-financing Accessed 23 Dec 2019.

[12] Cookson MS, Herr HW, Zhang ZF, Soloway S, Sogani PC, Fair WR (1997). The treated natural history of high risk superficial bladder cancer: 15-year outcome. J Urol.

[13] Bohle A, Brandau S (2003). Immune mechanisms in bacillus Calmette-Guerin immunotherapy for superficial bladder cancer. J Urol.

[14] Biot C, Rentsch CA, Gsponer JR, Birkhäuser FD, Jusforgues-Saklani H, Lemaître F, et al. Preexisting BCG-specific T cells improve intravesical immunotherapy for bladder cancer. Sci Transl Med. 2012;4:137ra72. 10.1126/scitranslmed.3003586. 10.1126/scitranslmed.3003586 22674550

[15] Niwa N, Kikuchi E, Matsumoto K, Kosaka T, Mizuno R, Oya M. Purified protein derivative skin test reactions are associated with clinical outcomes of patients with nonmuscle invasive bladder cancer treated with induction bacillus Calmette-Guérin therapy. Urol Oncol*.* 2018;36:e15–77.e21. 10.1016/j.urolonc.2017.10.005 29097085

[16] Martínez-Pineiro JA, Flores N, Isoma S, Solsona E, Sebastián JL, Pertusa C (2002). Long-term follow-up of a randomized prospective trial comparing a standard 81 mg dose of intravesical bacille Calmette-Guérin with a reduced dose of 27 mg in superficial bladder cancer. BJU Int.

[17] Chiou MY, Hsu BH, Huang YF, Chang HC. Assessment and perspective of BCG-vaccination policy in Taiwan. https://www.cdc.gov.tw/En/EpidemicTheme/Detail/hQ4XhaZAzUmNe2ksY4tjMA?archiveId=IDW4eMbqTQN1DaU4o8IaPg Accessed 03 Jun 2020.

[18] Lamm DL (1985). Bacillus Calmette-Guerin immunotherapy for bladder cancer. J Urol.

[19] Herr HW, Pinsky CM, Whitmore WF, Sogani PG, Oettgen HF, Melamed MR (1985). Experience with intravesical bacillus Calmette-Guerin therapy of superficial bladder tumors. Urology..

[20] Herr HW, Laudone VP, Whitmore WF (1987). An overview of intravesical therapy for superficial bladder tumors. J Urol.

[21] Lamm DL (2000). Efficacy and safety of bacille Calmette-Guerin immunotherapy in superficial bladder cancer. Clin Infect Dis.

[22] Krajewski W, Matuszewski M, Poletajew S, Grzegrzolka J, Zdrojowy R, Kolodziej A (2018). Are there differences in toxicity and efficacy between various bacillus Calmette-Guérin strains in bladder cancer patients? Analysis of 844 patients. Urol Int.

